# Evaluation of Microbial Transplantation from High-Productivity Soil to Improve Soybean Performance in Less Productive Farmland

**DOI:** 10.3390/microorganisms13061177

**Published:** 2025-05-22

**Authors:** Danilo Tosta Souza, Aurélio Carneiro Soares Moreira, Hélio Danilo Quevedo, André May

**Affiliations:** 1 EMBRAPA Environment, Brazilian Agricultural Research Corporation, Jaguariúna 13918-110, SP, Brazil; hd.qvdo@gmail.com (H.D.Q.); andre.may@embrapa.br (A.M.); 2 NOOA Agricultural Science and Technology, Patos de Minas 38700-970, MG, Brazil

**Keywords:** agricultural productivity, beneficial bacteria, crop yield, inoculant application, microbial consortium, rhizosphere microbiome

## Abstract

Microbial transplantation represents a sustainable strategy to address productivity gaps in agricultural soils by transferring microbiomes that enhance nutrient cycling, pathogen suppression, and stress tolerance. This study evaluates whether probiotic consortia from high-yield soybean soils (donor soil) could improve crop performance in less productive fields (recipient soil). We developed a host-adapted inoculant from soybean rhizospheres grown in donor soil and applied it to seeds at five concentrations (0.25–10 g/kg seed) in recipient soil, with untreated controls for comparison. To assess crop-specific microbial recruitment, we prepared a parallel bean-derived inoculant under identical conditions. Through 16S rRNA sequencing and growth/yield analysis, we found the following: (1) Distinct bacteriome assemblies between soybean- and bean-derived inoculants, confirming host specificity; (2) Successful enrichment of beneficial taxa (*Enterobacteriaceae* increased by 15–22%, *Rhizobiaceae* by 7–12%) despite native community resilience; and (3) Consistent yield improvement trends (4.8–6.2%), demonstrating potential to bridge productivity gaps. These results show that transplanted microbiomes can effectively modulate rhizosphere communities while maintaining ecological balance. This work establishes a scalable approach to address soil productivity limitations through microbiome transplantation. Future research should optimize (a) inoculant composition for specific productivity gaps; (b) delivery systems; and (c) compatibility with resident microbiomes, particularly in systems where niche-specific processes govern microbial establishment.

## 1. Introduction

Crop productivity is intrinsically linked to soil health, with beneficial microorganisms playing a critical role in promoting plant growth and resilience [1]. In high-performing agricultural soils, diverse and robust microbial communities facilitate nutrient cycling, suppress pathogens, and enhance plant stress tolerance, contributing to greater productivity [2,3,4]. Conversely, soils with diminished microbial diversity or an imbalance in beneficial strains often experience reduced productivity and ecosystem stability, as microbial dysfunction can favor the occupation of niches by pathogenic organisms, despite some functional redundancy within soil communities [5,6,7,8,9,10,11].

Despite decades of intervention, productivity gaps between high-yield and marginal farmlands persist. Traditional approaches, such as chemical fertilization, often fail to sustain long-term soil health due to nutrient leaching and disruption of the soil microbiome [6], while crop rotation alone cannot fully overcome microbial deficiencies in degraded soils [8]. These limitations highlight the urgent need for ecological alternatives capable of rebuilding soil functionality.

To address this agricultural productivity gap, microbial transplantation has emerged as a promising strategy, enabling the transfer of robust microbial consortia from healthy, high-performing soils to degraded or less productive areas, with studies demonstrating its success in restoring disease-suppressive soils through whole-community transfer [5]—a feat unattainable by single-strain inoculants prone to competitive exclusion [12]. Field studies in crops such as rice [13] and wheat [14] have demonstrated significant yield improvements following microbial transplantation. Indeed, this technique restores critical microbial functions, improving soil fertility and crop performance [1,15,16]. For instance, microbial consortia sourced from the rhizosphere of healthy plants have been shown to enrich soils with beneficial bacteria, such as Gammaproteobacteria, which contribute to plant protection and nutrient acquisition [5,11,17]. Similarly, soybean plant-derived microbial inoculants have demonstrated the ability to enhance crop yields and alleviate oxidative stress by positively influencing the plant microbiome [17,18,19,20,21,22].

The concept of microbial transplantation, originally explored in biomedicine and microbial ecology, now offers a scalable and sustainable solution for agricultural soil restoration [23]. By introducing complex microbial communities from donor to recipient soils, this method re-establishes microbial balance, promotes plant health, and restores fertility while providing natural disease resistance [1,11]. Advances in genomic sequencing, bioinformatics, and biotechnology further enhance the precision and efficacy of this approach [2]. High-throughput 16S rRNA sequencing enables the identification of keystone taxa [2], while metagenomic tools facilitate the design of consortia targeting specific functional traits [18]. These technologies address prior limitations by (1) mapping host–microbe interactions critical for transplantation success [16]; and (2) predicting ecological outcomes through network analysis [15], thereby optimizing both ecological and economic benefits.

Despite these advancements, challenges remain in effectively delivering microbial consortia. Integrating these inoculants with existing agricultural inputs, such as fertilizers, requires further research to maximize their efficacy [24,25]. Moreover, traditional single-species inoculants are often less effective than complex consortia capable of addressing multiple soil health challenges [12,26]. Supposedly, specialist microbes, strains well-adapted to the plant system, emerge as a solution to improve inoculant effectiveness, as they are more likely to establish in the rhizosphere, interact beneficially with native communities, and provide targeted benefits to plant growth and health [1,3,4,7,16,18,27].

Building on these principles, the present study develops a microbial consortium inoculant derived from the rhizosphere and rhizoplane of soybean plants grown under controlled mesocosm conditions using soil from a high-productivity soybean field. The objective was to determine whether these well-adapted microbial consortia could enhance seedling growth in fields with historically lower soybean productivity. Based on the principle that microbial diversity and abundance are key drivers of soil fertility [1,6,8,9,18], we hypothesized that transferring these consortia from productive to less productive soils would increase the abundance of beneficial microbial groups, thereby promoting seedling development.

To test this hypothesis, we characterized the soybean-associated bacteriome using 16S rRNA gene sequencing and evaluated the impact of microbial inoculation on rhizosphere composition and seedling growth. Our findings show that microbial transplantation from high-productivity areas can modestly improve yields and selectively alter the rhizosphere composition of less productive soils, without disrupting the overall microbial community structure. However, the effectiveness of microbial inoculants depends on environmental factors, soil properties, and interactions with native microbial communities. This study highlights the potential of microbial transplantation as a sustainable agricultural strategy while underscoring the need for further research into delivery mechanisms and interactions with native microbes to optimize its application in diverse agricultural systems.

## 2. Materials and Methods

### 2.1. Soil Sampling

Soil samples were collected in two agricultural fields. The first one is located in the municipality of Castro (24°47′57.4″ S and 49°53′16.7″ W), in the state of Paraná (PR), Brazil. The predominant soil type is Inceptisol (Soil Classification System), and the climate is classified as Cfb (subtropical humid with mild summers—Köppen classification) with an annual average air temperature of 16.8 °C and precipitation of 1553 mm. In this agricultural field, a no-till system was established more than 40 years ago. Crop rotation in this area includes soybeans, corn, wheat, and oats plus an annual application of swine manure. This first area was here considered a field of high soybean productivity, with an average of 5100 kg ha^−1^.

The second field is located in the municipality of Patos de Minas (18°52′31.5″ S and 46°37′45.1″ W), in the state of Minas Gerais, Brazil. The predominant soil type is Oxisols, and the climate is classified as Aw (tropical savanna climate with dry winters) with an annual average air temperature of 21.1 °C and precipitation of 1400 mm. In this area, a no-till system was established 42 years ago, and crop rotation includes soybeans and corn. Due to historically lower agricultural productivity compared to the first field (4320 kg ha^−1^), soil samples from this area were classified as a less productive soybean farmland.

**Figure 1:**
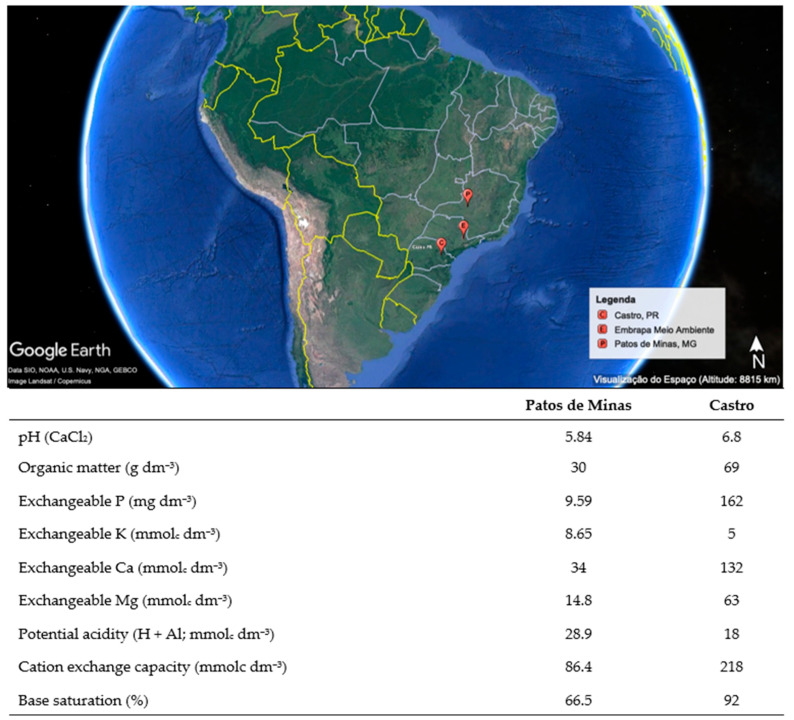
Image sourced from Google Earth showing the sampling locations and soil chemical properties of high-productivity soybean fields in Castro, PR, and low-productivity fields in Patos de Minas, MG. The map indicates Embrapa Meio Ambiente, where greenhouse experiments were conducted.

Soil samples were taken after harvesting in 2018. For this, the litter layer was first removed and then soil samples were taken from the 0–20 cm topsoil layer and determined as bulk soil. Rhizosphere soil samples from many soybean plants were also collected. In total, 70 soil samples (35 bulk soil and 35 rhizosphere soil; ~1.5 kg of soil per sample) were collected in a zigzag pattern across an area of 2 ha totaling 100 kg of soil collected from the first site. The second site (less productive field) was sampled following identical procedures to Site 1, though with a reduced sample number. All samples from both sites were transported within 8 h in sterile, thermally insulated stainless steel containers maintained at ambient temperature (25 ± 2 °C, monitored with calibrated data loggers) to ensure microbial integrity during transit to the Brazilian Agricultural Research Corporation (Embrapa Meio Ambiente), located in Jaguariúna, São Paulo, Brazil. Upon arrival, samples were immediately stabilized at 20 °C and processed within 48 h. Figure 1 provides detailed information on the main chemical properties of the soils and the localization of the sampling sites.

### 2.2. Construction of the Clonal Garden and Inoculant Preparation

In the laboratory, soil samples from a highly productive soybean field (bulk and rhizosphere soil) were mixed to create a homogeneous sample, which served as the donor soil for the total intraspecific microbial community in a mesocosm bioassay. Both rhizospheric and bulk soil microbiomes are critical to plant health and agricultural productivity [28]. Consequently, we combined these to capture the complete microbial community for formulating a specific consortium adapted to the plant environment and aimed at enhancing soybean productivity.

This experiment, referred to as the “clonal garden”, involved the multiplication and natural recruitment of microbial biomass within the soybean rhizosphere under controlled environmental conditions. The study was conducted in greenhouse seedling benches using the soybean cultivar TEC 7022 IPRO. Each seedling bench (2 m in length, 1 m in width, and 0.3 m in depth) was filled with a reactional mixture of donor soil. Soybean seeds (100 seeds per m^2^) were sown, and two identical seedling benches were established as technical replicates, each containing 600 L of standardized substrate.

The substrate was homogenized using a concrete mixing machine and consisted of five parts expanded clay to one part by volume of peat (SEDA line of the Legro group, Helmond, The Netherlands). To this, 25 g of Potasil (Yoorin group, Pocos de Caldas, Brazil), 25 g of phosphate fertilizer from Yoorin, and 2 g of dolomitic limestone were added. Donor soil (2 kg per m^2^ of the clonal garden) was broadcast onto the final mixture in the seedbeds.

The experiment was maintained at 28/19 °C (day/night) with a 12 h photoperiod. Soil moisture in the clonal garden was adjusted regularly using an aqueous solution prepared by inundating 10 kg of donor soil with water, vigorously agitating the mixture, decanting it for 2 min, and filtering through a clean cloth. The resulting liquid, enriched with microbes and nutrients, was applied via fertigation at a rate of 100 mL per m^2^.

After 21 days, the roots of healthy soybean plants, containing the rhizoplane and rhizosphere, were harvested and ground into a homogeneous black powder. This powder, containing the microbial consortium, was dehydrated in a forced air-drying oven at 28 °C for 7 days. The resulting microbial consortium inoculant was stored in hermetically sealed plastic bags at room temperature for later use.

To confirm the specificity of the microbial consortium inoculant derived from soybean plants, a bean-based inoculant was included as a control. Prepared under identical conditions using the bean cultivar Campos Gerais, this control provided the basis for evaluating differences in bacterial composition between the two inoculants, as detailed in the next section.

### 2.3. Inoculant Application and Seedling Growth Measures

The field experiment was conducted on low-productivity soybean farmland in Patos de Minas, MG. The study employed a randomized complete block design (RCBD) with six treatments and six repetitions (i.e., six blocks, each containing all six treatments). Treatments included five doses of the microbial consortium inoculant (0.25, 0.50, 0.75, 1.00, and 10.0 g per kg of soybean seed) and a non-inoculated control. The 10.0 g/kg dose was included as an extreme test case to evaluate potential saturation effects or dose-dependent responses, given that lower doses (0.25–1.00 g/kg) reflect typical agronomic ranges of biofertilizers. Each experimental unit consisted of five 6 m rows spaced 0.5 m apart (total area 15 m^2^), with the central three rows (excluding 0.5 m at each end) serving as the 7.5 m^2^ useful area to minimize edge effects. Standard crop management practices included fertilization with 100 kg ha^−1^ of MAP applied at planting and 400 kg ha^−1^ of a 30-00-20 (N-P_2_O_5_-K_2_O) formulation applied at the V4 growth stage. Weed, insect, and disease management followed Embrapa’s recommendations to ensure these factors did not limit the experiment.

The microbial consortium inoculant was then transferred to soybean seeds to evaluate its efficacy in seedling growth and impact on the rhizosphere when grown in an agriculture field with a history of low soybean productivity. The inoculant was applied to the seeds before sowing by microbiolization, according to the proposed doses. The inoculant was dispersed in 100 mL of water for each kg of soybean seeds, being applied by spraying and mixing in a concrete mixer. The microbiolized seeds were stored for a period of 2 h to ensure greater contact between microbes and seeds, and afterward, they were immediately sown. The rhizosphere microbiome was collected 90 days after planting in the field, at the R_2_ flowering stage. Grains were harvested to measure agricultural productivity at the end of the soybean crop cycle from a 7.5 m^2^ area in the central part of the plot.

In addition to assessing grain yield in open fields, seedling growth parameters (plant height, root and shoot dry mass) were evaluated in a greenhouse experiment. The experimental setup involved growing soybean seeds in soil from the field with a history of low soybean productivity and comparing it to the same soil treated with varying doses of soybean-based inoculant applied to microbiolized seeds. This greenhouse experiment was carried out for 21 days in 5 kg pots using natural soil (not autoclaved). The data obtained for seedling growth parameters were compared using one-way analysis of variance (ANOVA) with the F test applied at a probability level of 10%, and grain yield data were compared using regression analysis as described in the Supplementary Materials. All statistical analyses were performed using R software version 4.0.3.

### 2.4. DNA Extraction and Sequencing

Total DNA from each soil sample was extracted using the DNeasy Power Lyzer Power Soil DNA Isolation Kit (Qiagen Inc., Valencia, CA, USA) following the manufacturer’s instructions. DNA quality and quantity were assessed using NanoDrop 1000 spectrophotometry (Thermo Scientific, Waltham, MA, USA) and 1% sodium boric acid agarose gel electrophoresis.

Taxonomical profiling was performed using amplicon sequencing targeting the V3-V4 region of the bacterial 16S rRNA gene. Sequencing was conducted at the GoGenetic Facility, located in the municipally of Curitiba, State of Paraná, Brazil. For RNA-seq analysis, we selected the 1.00 g/kg seed inoculant dose based on its superior phenotypic performance—most notably, an increased flower count—observed during preliminary evaluations. Six biological replicates were collected, one from each experimental block, to capture field-scale variability. Identical sampling procedures were applied to both treated and untreated plants. A total of 25 DNA sample libraries were prepared to characterize the bacterial consortia: 4 libraries originating from the donor soil (native soil from the field with a history of high soybean productivity); 6 libraries of soybean inoculant produced by growing soybean plants in the donor soil (a bioproduct developed in this study using the clonal garden technique); 6 libraries from the soybean rhizosphere in farmland that is historically less productive (untreated recipient soil); 6 libraries from the soybean rhizosphere in less productive farmland treated with the donor soil-derived soybean inoculant (treated recipient soil); and 3 libraries of bean-based inoculant produced by growing bean plants in the donor soil (developed using the clonal garden technique).

The libraries were prepared using the Miseq Reagent Kit v3 (Illumina, San Diego, CA, USA), following the manufacturer’s instructions for the Illumina MiSeq platform (2 × 250 bp paired-end). Primers and reaction conditions used for amplification are described in Supplementary Materials.

### 2.5. Bacterial Community Analysis

The bacterial 16S rRNA sequences were processed using QIIME 2 (version 2020.11). First, the sequences were demultiplexed and quality control was carried out with DADA2 [29], using the consensus method to remove any remaining chimeric and low-quality sequences. Afterward, singletons, doubletons, chloroplast, and mitochondria sequences were removed from further analysis. Taxonomic classification was performed using a pre-trained classifier (silva-132-99-515-806-nb-classifier.qza) based on the SILVA database (v132) at 99% similarity [30], and the generated matrices were used for statistical analyses. The sequences are deposited in the NCBI database under the accession number PRJNA1259697.

To determine whether the bacterial community structure among treatments was significantly different from each other, we used Principal Coordinates Analysis (PCoA) followed by an ADONIS permutation-based statistical test in vegan-R with the weighted UniFrac distances matrix. The observed OTUs (Richness) and Shannon alpha diversity index were calculated based on the OTU table using base-R statistical packages and compared using Tukey’s HSD test. To compare the differential abundance of bacteria among treatments, the OTU table was used as input in the software Excel. *p*-values were calculated based on a two-sided Welch’s t-test with correction using Benjamini–Hochberg FDR.

## 3. Results

### 3.1. Definition of Terms

**Figure 2:**
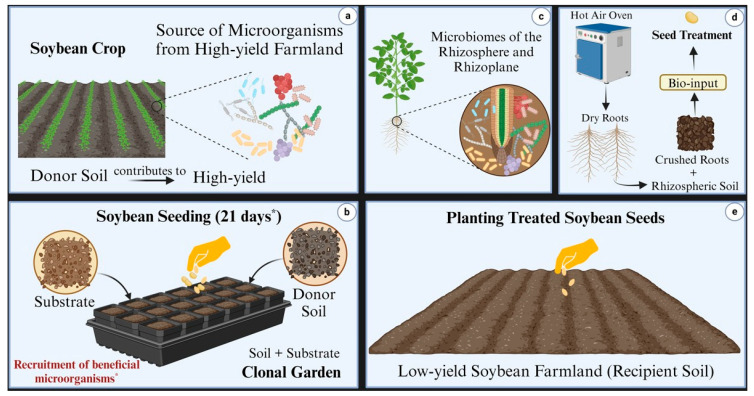
Illustrative scheme depicting the workflow for preparing inoculants. (**a**) Soil from high-yield farmland served as a donor of beneficial microbes. (**b**) Soybean plants were cultivated in a clonal garden containing donor soil to recruit key microbiomes, which formed the microbial consortium inoculant. The asterisk (*) indicates that plants grew for 21 days to recruit beneficial microorganisms. (**c**,**d**) The inoculants were prepared as a black powder by collecting, crushing, and dehydrating rhizosphere and rhizoplane samples. (**e**) Plants treated with the inoculant were grown in low-yield farmland to enhance productivity and microbial diversity. Results were compared with untreated plants.

In this study, donor soil refers to soil collected from a highly productive soybean farmland, serving as the source of beneficial microbial communities for inoculant preparation. Recipient soil refers to soil from less productive soybean farmland, targeted for microbial enrichment through inoculation. The terms treated and untreated soils refer to recipient soils that either received or did not receive the microbial consortium inoculant, respectively. Similarly, treated and untreated plants refer to soybean plants grown in these respective soils. The terms bean inoculant and soybean inoculant refer to microbial preparations derived from the rhizosphere and rhizoplane microbiomes of bean and soybean plants, respectively, collected from the donor soil. These inoculants were developed using a clonal garden strategy to ensure consistency and standardization, facilitating the inoculation of probiotic microbial strains as a consortium. Figure 2 presents the workflow for preparing inoculants using the clonal garden technique and transferring microbial consortia from high-productivity to less productive soybean farmland.

### 3.2. Alpha-Diversity Analysis

**Figure 3:**
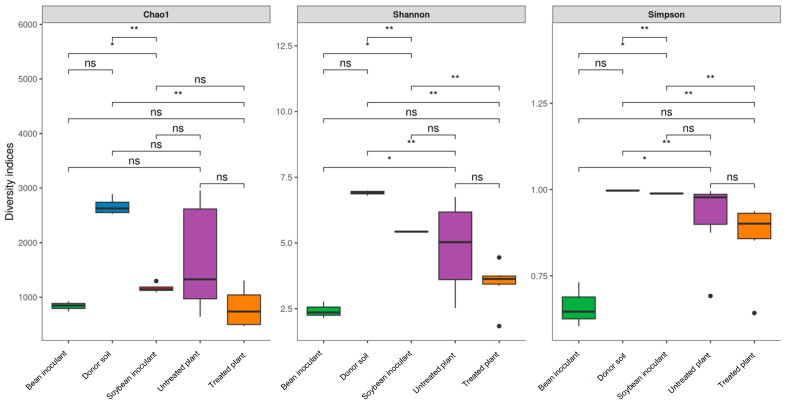
Alpha-diversity measures (Chao1, Shannon, and Simpson indices) of bacterial 16S rRNA amplicons across different sample types: donor soil (highly productive soybean farmland soil), soybean inoculant (microbial preparations from the rhizosphere and rhizoplane of soybean cultivated in donor soil), bean inoculant (microbial preparations from the rhizosphere and rhizoplane of bean cultivated in donor soil), untreated plant (soybean rhizosphere from a less productive field without soybean inoculant application), and treated plant (soybean rhizosphere from a less productive field with soybean inoculant application). Asterisks denote significant differences between treatments (Tukey’s test, *p* < 0.05 *, *p* < 0.01 **). “ns” indicates non-significant differences.

The alpha-diversity analysis revealed distinct bacterial characteristics among the inoculants, donor soil, and recipient soils. The soybean inoculant exhibited significantly lower species richness (Chao1, *p* = 0.003) and diversity (Shannon, *p* = 0.008) than the donor soil (Figure 3). Conversely, species richness between the donor soil and untreated plants grown in the recipient soil was statistically similar (Chao1, *p* = 0.21). However, differences in diversity, as indicated by the Shannon (*p* = 0.015) and Simpson (*p* = 0.021) indices, were evident. These discrepancies likely resulted from variations in edaphoclimatic conditions, as the soils sampled from different regions exhibited limited species overlap. Moreover, the higher diversity observed in the donor soil may be attributed to its composition, which included homogenized bulk soil and soybean rhizosphere samples collected from high-productivity fields. In contrast, the untreated plants represented rhizosphere communities derived exclusively from less productive soils.

Significant disparities in richness (Chao1, *p* = 0.008) and diversity (Shannon, *p* = 0.013) indices were also observed between the soybean and bean inoculants. Interestingly, the microbial communities inherent to inoculant constituents, such as peat-derived microbes, minimally impacted the bacterial composition. These findings emphasize the dominant role of host plants in shaping bacterial communities, even when grown under identical soil conditions.

No significant differences in richness (Chao1, *p* > 0.40) or diversity (Shannon/Simpson, *p* > 0.35) were observed between: (1) soybean inoculant and untreated groups, or (2) treated versus untreated plants (Figure 3). While the inoculant itself showed higher diversity (Shannon *p* = 0.008; Simpson *p* = 0.012) than field samples, it did not significantly alter overall rhizosphere diversity (all *p* > 0.05). Notably, both untreated and treated samples demonstrated increased variability in richness and diversity, as illustrated by alpha-diversity graphs (Figure 3). This variability likely reflected the effects of spatial and soil heterogeneity in open agricultural fields, which differentially shaped microbial dispersion across microhabitats.

### 3.3. Beta-Diversity and Taxonomic Composition

**Figure 4:**
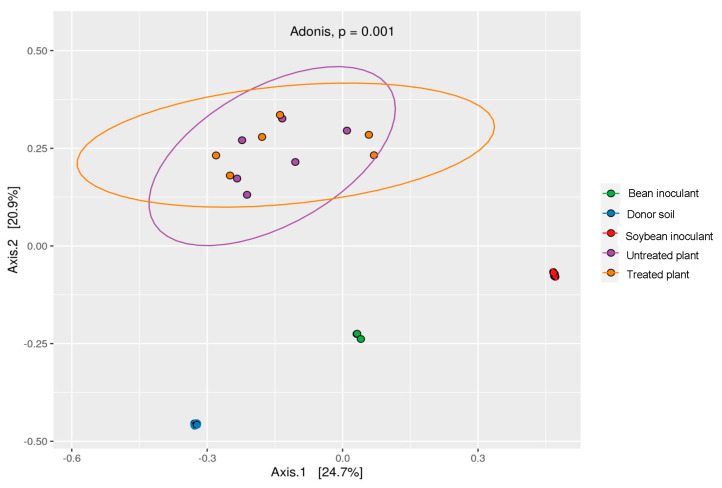
Principal Coordinate Analysis (PCoA) of bacterial communities at 99% similarity, based on 16S rRNA sequences using unweighted UniFrac distances. ADONIS test reveals distinct differences between donor soil and inoculants, with similarities between untreated and treated plants.

Principal Coordinate Analysis (PCoA) demonstrated significant compositional differences between donor soil and inoculant samples (PERMANOVA: *p* = 0.001), with soybean and bean inoculants forming distinct clusters (*p* = 0.003) that reflected host-specific recruitment during preparation. In contrast, field-grown plants showed remarkable community stability, with no significant differences between treated and untreated groups (*p* = 0.34; Figure 4). Beta-diversity metrics quantitatively supported these patterns: weighted UniFrac distances were 4-fold greater between donor soil and field plants (0.72 ± 0.03) than between treated and untreated plots (0.18 ± 0.02). Variance partitioning confirmed soil origin (donor vs. recipient) as the dominant structuring factor (R^2^ = 0.38, *p* = 0.001), while inoculation accounted for minimal variation (R^2^ = 0.05, *p* = 0.21). Together, these results demonstrate that while the clonal garden technique successfully generated host-specific inoculants, their application failed to enhance overall rhizosphere diversity in treated plants.

**Figure 5:**
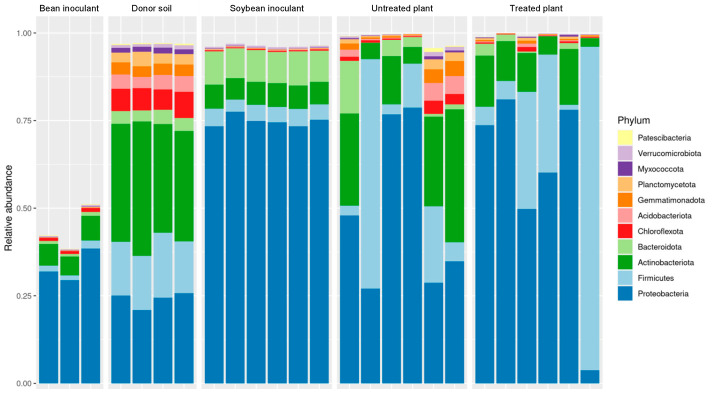
Taxonomic assignments at the phylum level showing the relative abundance (%) of the 16S rRNA gene sequences from different sample types: *bean inoculant* (microbial preparations from the rhizosphere and rhizoplane of bean cultivated in donor soil), *donor soil* (highly productive soybean farmland soil), *soybean inoculant* (microbial preparations from the rhizosphere and rhizoplane of soybean cultivated in donor soil), *untreated plant* (soybean rhizosphere from a less productive field without soybean inoculant application), and *treated plant* (soybean rhizosphere from a less productive field with soybean inoculant application).

In Figure 5, despite some proportional differences in group abundances, bacterial profiles in untreated and treated samples exhibited similarity, as previously demonstrated. Untreated samples contained an average of 29 phyla, with Proteobacteria (49%), Actinobacteriota (19%), Firmicutes (18%), Bacteroidota (4%), Acidobacteriota (2%), Gemmatimonadota (2%), Planctomycetota (1%), and Chloroflexota (1%), each accounting for more than 1% of operational taxonomic unit (OTU) relative abundance. Similarly, treated samples averaged 26 phyla, dominated by Proteobacteria (58%), Firmicutes (28%), Actinobacteria (10%), and Bacteroidota (1%), each exceeding 1% of OTUs. The phyla detected at less than 1% in both untreated and treated samples were categorized as components of the rare biosphere.

The soybean-derived inoculant exhibited a distinct metataxonomic profile compared to the donor soil. Proteobacteria predominated in the inoculant accounting for 77% of observed OTUs across 21 bacterial phyla. This was in sharp contrast to the donor soil, where Proteobacteria represented only 24% of OTUs across a more diverse spectrum of 30 phyla. Other significant phyla in the soybean inoculant included Bacteroidota (9%), Actinobacteriota (7%), and Firmicutes (5%). Conversely, the donor soil exhibited higher relative abundances of Chloroflexota (6%), Acidobacteriota (4%), Gemmatimonadota (3%), Planctomycecota (3%), and Myxococcota (1%), which collectively contributed less than 1% of the reads in the soybean inoculant.

On the other hand, the bean-based inoculant was similarly dominated by Proteobacteria (75%) but included a higher proportion of Actinobacteriota (14%) alongside Firmicutes (4%) and Bacteroidota (2%). This inoculant comprised 22 bacterial phyla, including Nitrospinota, a group absent in the donor soil but detected as part of the rare biosphere within the inoculant. The appearance of Nitrospinota suggests its origin from external sources, such as the peat used in the formulation or the seeds of the bean cultivar Campos Gerais. However, its absence in the donor soil and other samples may also reflect technical limitations in sequencing, particularly in detecting low-abundance taxa associated with the rare biosphere.

In this study, the methodology involving a clonal garden and inoculant preparation from the soybean rhizosphere and rhizoplane revealed the absence of several bacterial groups in the inoculants, including Methylomirabilota, Dormibacterota, Elusimicrobiota, candidatus 4484-113, Deinococcota, Tectomicrobia, Halobacteriota, Fibrobacterota, and Eisenbacteria. This absence was likely due to the rhizosphere’s recruitment of a highly specialized bacterial community predominantly composed of Proteobacteria, Bacteroidota, Actinobacteriota, and Firmicutes.

Interestingly, many phyla undetected in the inoculants were naturally present in both the donor and recipient soil samples. However, certain groups, such as Tectomicrobia, Halobacteriota, Fibrobacterota, and Eisenbacteria remained undetected in the recipient soil. These findings suggest that the phyla absent from both the recipient soil and inoculants may naturally occur only in the donor soil, as soybean plants failed to recruit these groups in the rhizosphere.

### 3.4. Deeper-Level Taxonomic Analysis

**Figure 6:**
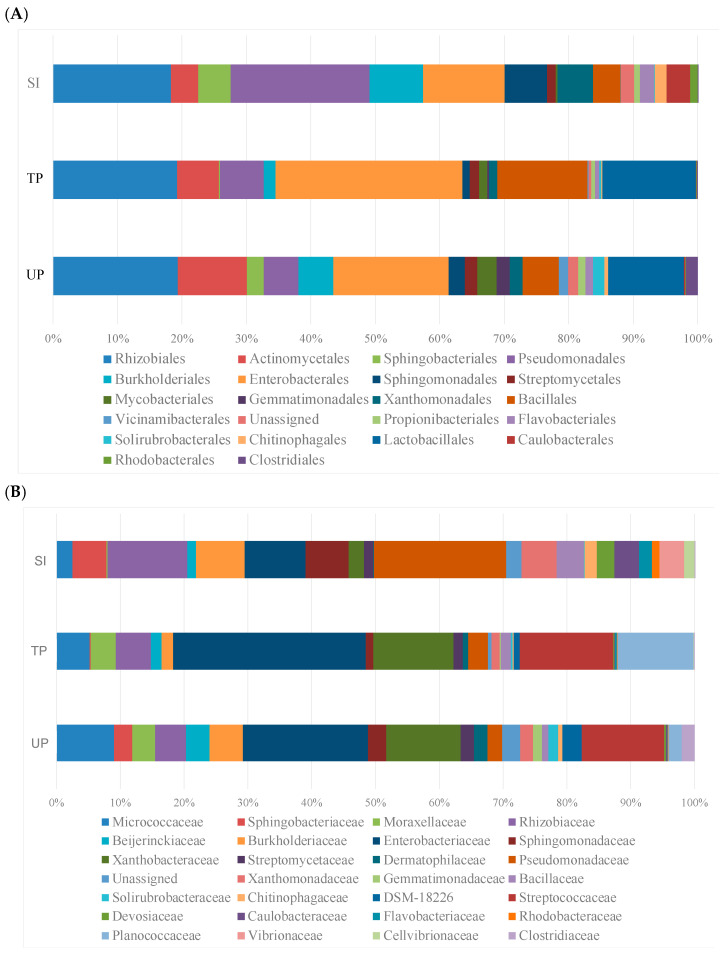
Distribution of the most abundant bacterial at the order (**A**) and family (**B**) levels based on the 16S rRNA gene sequences from different sample types: soybean inoculant, SI (microbial preparations from the rhizosphere and rhizoplane of soybean cultivated in donor soil); treated plants, TP (soybean rhizosphere from a less productive field with soybean inoculant application); and untreated plants, UP (soybean rhizosphere from a less productive field without soybean inoculant application).

Taxonomic analysis at the order level revealed statistically significant changes in specific bacterial taxa following inoculation (*p* < 0.05 for all reported groups), despite overall community stability (PERMANOVA *p* = 0.34). Key taxonomic orders driving the differentiation of the samples, as shown in Figure 6A, include Rhizobiales, Enterobacterales, Pseudomonadales, Actinomycetales, Streptomycetales, Bacillales, Lactobacillales, Burkholderiales, Propionibacteriales, Sphingobacteriales, and Xanthomonadales, all with relative abundances exceeding 5% across the dataset.

More detailed family-level analysis (Figure 6B) demonstrated specific enrichment of plant-growth-promoting taxa in treated plants, most notably *Enterobacteriaceae* (22.3% increase, *p* = 0.008)—known for nitrogen fixation and phytohormone production—and *Rhizobiaceae* (15.7% increase, *p* = 0.012), which are essential for soybean nodulation. These beneficial increases coincided with reductions in native soil-adapted taxa, including *Micrococcaceae* (11.2% decrease, *p* = 0.021) and stress-tolerant *Sphingobacteriaceae* (8.5% decrease, q = 0.038), suggesting competitive displacement of some native specialists. The treatment also enhanced other functionally important families like Pseudomonadaceae, *Xanthobacteraceae*, *Moraxellaceae*, *Bacillaceae*, *Streptococcaceae*, and *Planococcaceae* (*p* < 0.05), while reducing abundance of *Beijerinckiaceae* and *Burkholderiaceae.*

The selective establishment of inoculated beneficial taxa without broader community disruption implies a balanced trade-off: the treatment successfully enriched key functional groups while the resident microbiome’s resilience prevented complete restructuring. The maintenance of the overall community structure despite these taxon-specific changes highlights two key findings: (1) the remarkable resilience of established soil microbiomes after 90 days of succession and (2) the ability to selectively enhance beneficial taxa without disrupting critical soil functions.

### 3.5. Greenhouse Experiment and Agricultural Productivity in the Field

A greenhouse experiment evaluated the performance of soybean plants after the incorporation of soybean inoculant sourced from highly productive to less productive soybean fields. The results indicate no significant differences in germination percentage, plant height, root dry mass, or shoot dry mass between treated and untreated plants (Table 1). Visual observations supported these findings, suggesting that transplanting microbiomes from a high agricultural productivity area to a less productive area, using the study’s methodology, did not effectively promote soybean growth. It is worth noting, however, that the study’s duration (21 days post-inoculation) may not capture short- or long-term effects on soil microbiome and plant growth.

**Figure 7:**
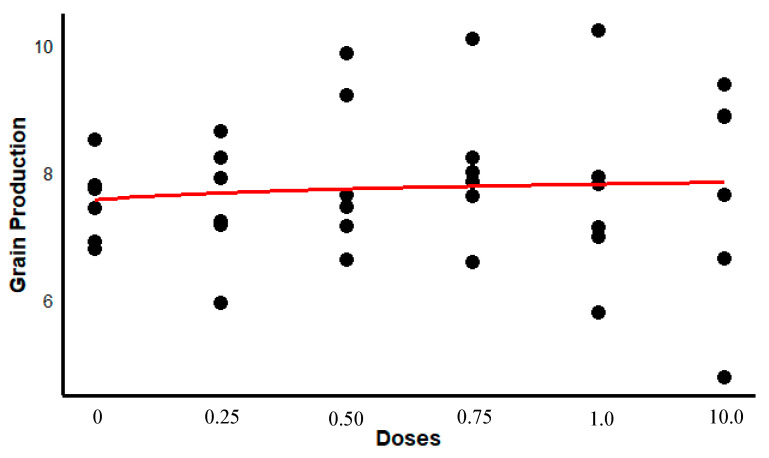
Response curve showing soybean grain yield as a function of microbial consortium inoculant applied to seeds before sowing. Five different dosages of inoculant (0.25, 0.50, 0.75, 1.00, 10.0 g of inoculant per Kg of soybean seed) were tested (treated plants), along with a control group without inoculant (untreated plants).

In terms of soybean agricultural productivity (kg ha^−1^), although non-significant, a marginal increase of 240 kg ha^−1^ (4.8–6.2%, *p* = 0.08) post-transplantation was noted in variable dosages, indicating potential benefits on crop yield (Figure 7).

## 4. Discussion

This study addresses current agricultural challenges by leveraging microbiological interventions to promote sustainable crop production. Specifically, we aimed to improve soil health and plant productivity by transferring microbial consortia from the rhizosphere and rhizoplane of soybeans grown in high-productivity fields to lower-productivity farmland. As highlighted by Moretti et al. [4], inoculation significantly impacts bacterial communities, with more pronounced effects on rhizofertility than on archaeal or fungal communities. Bacteria were prioritized in this study due to their sensitivity to microbiological interventions and their role as key indicators of soil health and rhizosphere modulation. They also dominate crucial plant-growth-promoting functions—such as nitrogen fixation and phosphorus solubilization—respond rapidly to inoculation and have well-established detection methods. Although archaea and fungi also contribute to soil health, their slower dynamics made bacteria more suitable for this proof-of-concept study evaluating the immediate effects of microbial transfer. Using 16S rRNA gene sequencing, we profiled bacterial communities in inoculated and non-inoculated plants, as well as in the microbial consortia used for inoculation, providing a robust framework to assess inoculation efficacy in modulating the soybean rhizosphere.

The rhizosphere is a dynamic environment shaped by interactions between specialist and generalist microbes, both of which are critical for plant health and soil function [1,7,16,28]. Specialist taxa, such as soybean-associated *Rhizobiaceae*, often form host-specific mutualisms, while generalists like *Pseudomonadaceae* thrive across diverse environments due to their metabolic versatility [1,10,28]. In our study, bacterial community composition and diversity indices differed significantly between the two soils, likely due to contrasting edaphoclimatic conditions [31,32]. These conditions influenced the success of microbial transfer: *Rhizobiaceae* abundance increased by 15.7% in inoculated soils (*p* = 0.012), likely driven by host-specific interactions, while generalist taxa showed variable establishment. Soil origin explained a substantial portion of microbial variance (R^2^ = 0.38, *p* = 0.001), while inoculation accounted for a smaller effect (R^2^ = 0.05), underscoring both the resilience of native microbial communities and the importance of matching inoculant traits to soil compatibility [33]

Traditional inoculants, typically composed of one or a few microbial strains, often yield inconsistent results due to the complexity and competitiveness of soil microbiomes [11,12]. In contrast, indigenous microbial consortia—microbes naturally adapted to local soils and host plants—offer a promising alternative to enhance crop resilience and productivity [3,11,16,22]. This strategy has proven effective in diverse fields, including human health, ecosystem restoration, and soybean cultivation [3,5,11,23]. Following this approach, we employed the “clonal garden” technique to develop a microbial consortium specifically enriched for soybean-associated microbes from high-productivity soils. This method targets locally adapted microbial species, potentially offering advantages over more generalized native inoculants. Furthermore, combining the microbial material with peat and fertilizer may have introduced rare groups such as Nitrospinota, involved in nitrite oxidation and nitrogen cycling [24,25,34]. However, this group was detected only in bean inoculants, highlighting rhizosphere selectivity.

Differences between bean- and soybean-derived inoculants likely reflect rhizosphere-specific recruitment processes shaped by plant genotype, root exudates, soil properties, and developmental stage [7,35,36]. Notably, the inoculant was derived from 21-day-old soybean plants, while rhizosphere samples in the recipient soils were collected at 90 days post-sowing. This temporal mismatch may have influenced microbial dynamics, as recruitment patterns evolve during plant development [32]. Although not directly tested, our findings suggest that future studies should align inoculation timing with plant phenology to maximize microbial establishment and function.

Consistent with Mendes et al. [1], our results reinforce the rhizosphere as a central interface for plant–microbe interactions. Transferring microbiota from high- to low-productivity soils modulated specific microbial taxa and led to modest improvements in plant performance. Similar outcomes have been reported in other crops: rice fields receiving microbiota from high-productivity soils showed improved nitrogen use efficiency [13], and wheat experienced 8–12% yield gains after rhizosphere transplantation [14]. Nevertheless, the variability in outcomes—including our own—highlights the importance of soil type, environmental context, and inoculant composition [4,11,17,27,36].

Contrary to expectations, treated plants did not fully reflect the diversity present in the inoculant, likely due to competitive exclusion and ecological filtering favoring native taxa [7,8]. For instance, although Proteobacteria dominated the inoculant, they showed limited establishment in the treated soils, emphasizing the selective nature of rhizosphere colonization [7]. While the inoculant enriched certain beneficial taxa, it did not significantly alter the overall community structure. This is supported by stable alpha-diversity indices (Shannon *p* = 0.38; Simpson *p* = 0.41), minimal beta-diversity shifts (PCo2 variance = 6.2%), and a high degree of shared ASVs (78%) between treated and control samples. These findings suggest that inoculants can enhance specific microbial groups while preserving core microbiome resilience over a 90-day period.

This pattern aligns with concepts from microbial invasion ecology, where introduced taxa often occupy narrow ecological niches without disrupting resident communities [37,38]. The observed “priority effect” allows introduced microbes to persist by exploiting underutilized metabolic functions without inducing competitive displacement [39]. Our data support the stochastic niche occupancy model [40], where introduced and native microbes coexist through niche partitioning, metabolic complementarity, and neutral interactions. These findings mirror observations in other cropping systems where successful inoculants supplement, rather than replace, indigenous microbiota [41]. Considering the temporal and spatial variability of field conditions, future research should prioritize stage-specific sampling to determine optimal inoculation windows—particularly early in development, when microbial communities may be more receptive to modulation.

Further taxonomic analysis revealed enrichment of beneficial bacterial families, such as *Enterobacteriaceae* and *Rhizobiaceae*, in inoculated rhizospheres. This agrees with previous studies showing targeted increases in growth-promoting genera like *Enterobacter*, *Pseudomonas*, and *Xanthomonas* [11]. However, concurrent declines in other microbial groups indicate that the inoculant exerted a selective rather than broad-spectrum effect on community composition. These findings are consistent with research suggesting that microbial inoculants shift microbial balances but do not necessarily confer universal benefits [7,42,43]. In our greenhouse experiment, no significant differences in seedling growth were observed between treatments, possibly due to the short trial duration (21 days), which may have been insufficient to detect plant-level impacts. Nevertheless, the marginal 6.2% increase in soybean yield observed under field conditions suggests that microbial transfers may confer agronomic benefits under more variable and stressful environments. Given that traditional plant breeding typically achieves annual yield gains of 1–2% [44], a single microbial intervention yielding over 6% is notable.

The contrasting results between greenhouse and field trials underscore the importance of environmental context in determining inoculant efficacy. In the controlled greenhouse setting, microbial activity may have been constrained by stable conditions. In contrast, the 90-day field trial introduced abiotic variability (e.g., temperature, moisture, and nutrient fluctuations), creating ecological niches that may have favored the activity and persistence of introduced microbes. The extended timeframe also allowed for more sustained interactions between the microbiome and plant physiology—critical elements often absent in short-term studies.

These results suggest that microbial inoculants act more effectively as buffers against environmental stress rather than as direct growth stimulants. This is consistent with meta-analyses showing that co-inoculation often yields modest or inconsistent results unless closely matched to specific environmental conditions [43]. Still, emerging evidence demonstrates considerable yield improvements in soybean and other crops when inoculants are ecologically tailored [4,22,29,45]. Our findings contribute to this growing body of evidence by demonstrating that intraspecific microbial transfers can enhance rhizosphere function and support crop performance in marginal soils.

Overall, our results highlight the importance of long-term, field-based studies that capture environmental variability across full crop cycles. They also suggest a dual mechanism: while soil fertility parameters establish the fundamental productivity potential, microbial consortia can provide incremental yield improvements by optimizing nutrient use efficiency and stress tolerance. This synergistic effect implies that microbial transplantation should be viewed as a complement to—rather than replacement for—balanced fertilization in low-fertility soils. Future research should focus on optimizing inoculant formulation, application timing, and delivery strategies—particularly for degraded or low-fertility soils. These efforts should be grounded in ecological principles such as niche complementarity and host–microbe specificity to improve inoculant persistence and function in diverse agroecosystems.

## 5. Conclusions

This study demonstrates the feasibility and potential of using host-adapted microbial consortia derived from high-productivity soybean soils to enhance crop performance in less fertile farmland. The inoculant selectively enriched beneficial bacterial families such as *Enterobacteriaceae* and *Rhizobiaceae*, which are associated with nutrient acquisition and nodulation. Importantly, these changes occurred without disrupting the broader rhizosphere microbial community, indicating a balanced integration with native soil microbiota. While short-term greenhouse trials showed limited plant growth responses, field experiments revealed a modest yield increase of 4.8–6.2%, suggesting that microbial transplantation may confer agronomic benefits under real-world environmental conditions. These findings support the concept that microbial inoculants can function more effectively as resilience-enhancing agents than as direct growth stimulants.

To fully realize the agronomic potential of microbiome transplantation, long-term, multi-site field trials should be conducted across diverse agroecological zones and cropping systems. Such trials should assess microbial persistence, functional stability, and crop performance across complete growth cycles, while also evaluating interactions with soil type, climate variability, and crop management practices. In particular, future work should explore the synchronization of inoculation with crop phenological stages to optimize plant–microbe interactions and improve microbial establishment. Furthermore, integrating microbial inoculants with existing fertilization strategies may offer synergistic benefits, particularly in nutrient-poor soils. Collectively, these efforts will be essential to refining inoculant composition, formulation, and delivery methods, thereby enhancing the ecological compatibility and efficacy of microbiome-based solutions for sustainable agriculture.

## Figures and Tables

**Table 1:** Effects of a microbial consortium inoculant, derived from soybean plants cultivated in a high-productivity field, on soybean development in less productive soil. Growth parameters, including germination rate, plant height, root dry mass, and shoot dry mass, were evaluated in a greenhouse pot experiment. Each treatment included six biological replicates (n = 6).

Seedling Properties	Inoculant Doses (g kg^−1^ of Seeds)					
	0.0	0.25	0.50	0.75	1.0	10.0
Germination (%)	93.3 ± 10.3	83.3 ± 15.1	73.3 ± 16.3	90.0 ± 11.0	90.0 ± 11.0	83.3 ± 23.4
Plant height (cm)	15.7 ± 1.4	15.7 ± 1.1	15.7 ± 1.5	16.0 ± 0.6	16.1 ± 1.1	15.3 ± 1.3
Root dry weight (g pL^−1^)	0.156 ± 0.012	0.171 ± 0.023	0.134 ± 0.044	0.155 ± 0.028	0.137 ± 0.028	0.150 ± 0.028
Shoot dry weight (g pL^−1^)	0.35 ± 0.03	0.37 ± 0.02	0.37 ± 0.03	0.38 ± 0.04	0.35 ± 0.06	0.36 ± 0.02

## Data Availability

The original contributions presented in this study are included in the article/Supplementary Materials. Further inquiries can be directed to the corresponding author.

## Supplementary Materials

The following supporting information can be downloaded at: https://www.mdpi.com/article/10.3390/microorganisms13061177/s1. File S1: Supplementary Material. References [46,47,48,49,50] are cited in the Supplementary Materials.
