# Supplementary material for: Evaluation of Microbial Transplantation from High-Productivity Soil to Improve Soybean Performance in Less Productive Farmland
Source: Microorganisms. 2025 May 22;13(6):1177. doi: 10.3390/microorganisms13061177 (PMC12195484; doi:10.3390/microorganisms13061177)
Supplement: Supplementary file 1 [file microorganisms-13-01177-s001.zip › microorganisms-3545185-supplementary.pdf]

Supplementary Material

Title: Evaluation of Microbial Transplantation from High-Productivity Soil to Improve Soybean Performance in a Less Productive Farmland

Authors: [1]Danilo Tosta Souza (Orcid 0000-0002-8093-6663); [2]Aurélio Carneiro Soares Moreira (Orcid 0009-0002-2121-158X); [1]Hélio Danilo Quevedo (Orcid 0000-0002-8334-5922) & [1]André May (Orcid 0000-0001-6157-7215)

Affiliation: 1Embrapa Meio Ambiente, Jaguariúna, São Paulo, 13918-110, CP 69, Brazil; 2NOOA Ciência e Tecnologia Agrícola, Patos de Minas, Minas Gerais, 38700-970, Brazil.

Corresponding author: Danilo Tosta Souza; danilo_tosta@hotmail.com

Material and methods

Response curve and statistical analysis

The data used in the present study were collected from a dataset that included the variables "Dose", representing different doses of a treatment applied to soybean samples, and "Grain", representing the measured response. To model the response of the "Grain" variable as a function of the "Dose" variable, a simple linear model was fitted using the lm function from the R software (Ihaka & Gentleman 1996). The equation of the fitted model is described as follows:

$$Grain = \beta_0 + \beta_1 \, x \, Dose + \epsilon$$

Where β_0 is the intercept, β_1 is the regression coefficient, and ϵ is the error term. To evaluate the model fit, a summary was generated using the summary function (modelo_linear) (Ihaka & Gentleman 1996). This summary provides detailed information on the model coefficients, p-values, R^2, and other indicators of model quality. From the fitted model, predicted values of the "Grain" variable for a sequence of doses were generated using the predict function (Ripley et al. 2021). These predictions allow the visualization of the expected response at different levels of treatment dose.

The dataset of soybean seedling development was preliminary analysed to check for normality of errors using the Shapiro–Wilk test (Shapiro and Wilk 1965) and for homoscedasticity of variances using Levene's test (Levene 1960). Subsequent data analysis was performed in R software (Core Team R 2020). The data were subjected to analysis of variance (ANOVA) with the application of the F test at a 10% probability level. The means were compared using the modified t-test (Least Significant Difference: LSD) with a significance level of $p \leq 0.10$.

Primers and reaction conditions used for amplification

The V3-V4 region of the bacterial 16S rRNA gene was amplified with region-specific primers (515F/806R) (Caporaso et al. 2011). Each 25 μL PCR reaction contained the following: 12.25 μL of nuclease-free water (Certified Nuclease-free, Promega, Madison, WI, USA), 5.0 μL of buffer solution 5× (MgCl2 2 Mm), 0,75 μL of solution of dNTP's (10 mM), 0,75 μL of each primer (515 YF 40 μM e 806 R 10 μM), 1.0 unit of Platinum Taq polymerase High Fidelity in concentration of 0,5 μL (Invitrogen, Carlsbad, CA, USA), and 2.0 μL of template DNA. Furthermore, a control reaction was performed by adding water instead of DNA. The conditions for the PCR reaction were as follows: 95 °C for 3 min, 35 cycles at 98°C for 20s, 55°C for 20s, and 72°C for 30s, and a final extension of 3 min at 72 °C.

After indexing, the PCR products were cleaned up using Agencourt AMPure XP – PCR purification beads (Beckman Coulter, Brea, CA, USA), according to the manufacturer's manual, and quantified using the dsDNA BR assay kit (Invitrogen, Carlsbad, CA, USA) on a Qubit 2.0 fluorometer (Invitrogen, Carlsbad, CA, USA). Once quantified, equimolar concentrations of each library were pooled into a single tube. After quantification, the molarity of the pool was determined and diluted to 2 nM, denatured, and then diluted to a final concentration of 8.0 pM with a 20% PhiX (Illumina, San Diego, CA, USA) spike for loading into the Illumina MiSeq sequencing machine (Illumina, San Diego, CA, USA).

References

Caporaso JG, Lauber CL, Walters WA, Berg-Lyons D, Lozupone CA, Turnbaugh PJ, Fierer N, Knight R (2011) Global patterns of 16S rRNA diversity at a depth of millions of sequences per sample. Proc Natl Acad Sci USA 108:4516-4522. https://doi.org/10.1073/pnas.1000080107

Core Team R, Team R. RC. R: a language and environment for statistical com- puting. 2020. 2021. R Foundation for Statistical Computing, Vienna, Austria.

Levene, H. (1960). Contributions to probability and statistics. Essays in honor of Harold Hotelling, 278, 292.

Ripley, B., Venables, W., Bates, D. M., Hornik, K., Gebhardt, A., & Firth, D. (2021). Package 'stats'. R Foundation for Statistical Computing. https://CRAN.R-project.org/package=stats

Shapiro SS, Wilk MB. An analysis of variance test for normality (Complete Samples). Biometrika. 1965;52:591.
